# Impact of Neoadjuvant Therapy on PD-L1 Expression in Triple-Negative Breast Cancer and Correlation with Clinicopathological Factors

**DOI:** 10.3390/diagnostics14232672

**Published:** 2024-11-27

**Authors:** Nevena Ilieva, Mina Pencheva, Hristo Hadzhiev, Desislava Tashkova, Elena Daskalova, Petar Georgiev, Sylvia Genova

**Affiliations:** 1 Department of General and Clinical Pathology, Faculty of Medicine, Medical University of Plovdiv, Bul. Vasil Aprilov 15A, 4000 Plovdiv, Bulgaria; desislava.tashkova@mu-plovdiv.bg (D.T.); sylvia.genova@mu-plovdiv.bg (S.G.); 2 Clinical Pathology Department, Complex Oncology Center Plovdiv, Bul. Vasil Aprilov 15A, 4000 Plovdiv, Bulgaria; 3 Department of Medical Physics and Biophysics, Faculty of Pharmacy, Medical University of Plovdiv, Bul. Vasil Aprilov 15A, 4000 Plovdiv, Bulgaria; mina.pencheva@mu-plovdiv.bg; 4 First Oncological Department, Complex Oncology Center Plovdiv, Bul. Al. Stamboliyski 2A, 4000 Plovdiv, Bulgaria; hristo.hadzhiev@abv.bg; 5 Department of Anatomy, Histology and Embryology, Faculty of Medicine, Medical University of Plovdiv, Bul. Vasil Aprilov 15A, 4000 Plovdiv, Bulgaria; elena.daskalova@mu-plovdiv.bg; 6 Faculty of Medicine, Medical University of Plovdiv, Bul. Vasil Aprilov 15A, 4000 Plovdiv, Bulgaria; pgio2000@abv.bg

**Keywords:** neoadjuvant therapy, PD-L1 expression, triple-negative breast carcinoma

## Abstract

Background: This study aims to deliver more insights on the impact of neoadjuvant treatment on Pd-L1 expression and to evaluate its correlation with clinicopathological factors. Methods: We reviewed 88 TNBC cases for the period 2021–2023. Data on age, tumor size, stage, and treatment were collected. Histological slides were assessed for subtype, grade, and TILs. A total of 48 received neoadjuvant treatment. HER2 and Ki67 were evaluated via immunohistochemistry. PD-L1 expression was tested on primary and residual tumors. Statistical analysis was performed using IBM SPSS (*p* < 0.05). Results: In this study, PD-L1 positive expression was found in 44.3% of primary tumors, with 52.9% of initially positive cases losing expression post-treatment. TILs were significantly higher in PD-L1-positive tumors (mean 41.79% vs. 27.55%, *p* = 0.001). A notable correlation was found between PD-L1 expression and Ki-67 proliferation index, with PD-L1-positive tumors having a median Ki-67 of 64.49 compared to 52.86 in negative cases (*p* = 0.015). Neoadjuvant immunotherapy led to a lower mean residual cancer burden (0.95 vs. 2.55, *p* = 0.002) compared to chemotherapy alone. Higher Ki-67 levels (≥50%) were associated with better treatment outcomes, showing a mean RCB score of 1.60 versus 3.16 for lower levels (*p* = 0.022). HER2-negative cases had a higher prevalence of favorable pathological response (54.5%) compared to HER2-low tumors (25%, *p* = 0.048), because of the strong correlation to high proliferative index. Conclusions: In conclusion, PD-L1 expression in TNBC shows significant discordance post-treatment, highlighting the need for routine testing and further research on predictive biomarkers.

## 1. Introduction

Triple-negative breast cancer (TNBC) is a particularly aggressive subtype of breast cancer characterized by the absence of estrogen receptors, progesterone receptors, and human epidermal growth factor receptor 2 (HER2). Representing approximately 10–20% of all breast cancer cases, TNBC is associated with a higher rate of metastasis and poorer prognosis compared to other breast cancer subtypes [1].

TNBC is a heterogenous group of tumors. Histologically, most of the tumors are of no special type (NST). However, certain histological forms of TNBC, like low-grade adenosquamous, low-grade adenoid-cystic, secretory, and mucoepidermoid carcinoma, have more favorable prognosis and may not necessitate systemic chemotherapy unless high-risk clinical features are present [2,3]. From a pathological point of view, TNBC is usually associated with higher stage, moderate or poor grading (G2/G3), and a high proliferative index (MYB-1/Ki-67) [4].

Current systemic treatment recommendations by the National Comprehensive Cancer Network (NCCN) include anthracycline- and taxane-based chemotherapy [5,6]. In patients carrying germline BRCA1/2 mutations, the use of platinum-based chemotherapy and Poly (ADP-ribose) polymerase (PARP) inhibitors is also approved [7,8]. The use of immunotherapy is advised for early-stage disease regardless of PD-L1 expression and in the metastatic setting of tumors with PD-L1 CPS ≥ 10 [9,10].

According to NCCN Guidelines, for second-line options in metastatic TNBC, the use of antibody–drug conjugates is also recommended—mainly sacituzumab govitecan [11] and trastuzumab deruxtecan in cases with HER2 expression being 1+ or 2+ with negative in situ hybridization [12,13].

From a molecular perspective, triple-negative breast cancer (TNBC) is a heterogeneous entity. This diversity was first characterized by Lehman and colleagues, who identified seven molecular subtypes of TNBC based on distinct gene expression profiles [14]. Subsequent research has refined these classifications into four primary molecular subtypes:

1. LAR (Luminal Androgen Receptor subtype): This subtype is marked by the expression of genes associated with the androgen receptor (AR), estrogen receptor (ER), ESR1, and other estrogen-regulated genes, despite demonstrating negative immunohistochemical expression for ER.
2. MES (Mesenchymal subtype): This subtype exhibits enrichment in genes implicated in cell motility and signaling pathways related to growth factors.
3. BLIS (Basal-like Immunosuppressed subtype): Characterized by downregulation of immunoregulatory pathways and dysfunction in DNA repair mechanisms, this subtype also shows expression of VEGFR and transcription factors from the SOX family.
4. BLIA (Basal-like Immunoactivated subtype): In contrast, this subtype is defined by the upregulation of immunoregulatory mechanisms and increased expression of STAT genes [15,16]

Ongoing research aims to develop targeted therapies tailored to these distinct molecular subgroups. For instance, approaches for the LAR subtype include the inhibition of CDK4/6, AR, and the PI3K/AKT/mTOR signaling pathways [14,17]. For the MES and BLIS subtypes, therapies may involve tyrosine kinase inhibitors (TKIs) that target EGFR and VEGFR pathways [17]. Lastly, potential treatments for the BLIA subtype encompass STAT inhibitors, cytokine receptor antibodies, and CTLA-4 inhibitors [18].

Recent advancements in immunotherapy for triple-negative breast cancer (TNBC) have significantly expanded treatment options and improved outcome for patients, particularly those with advanced or metastatic disease. Historically, TNBC has been one of the most challenging breast cancer subtypes to treat due to its lack of expression of estrogen, progesterone, and HER2 receptors, which makes it unresponsive to targeted therapies. However, the emergence of immune checkpoint inhibitors (ICIs) has provided new hope.

One of the most notable advancements has been the approval of pembrolizumab (an anti-PD-1 monoclonal antibody) for use in combination with chemotherapy for patients with locally advanced or metastatic TNBC, especially those whose tumors express PD-L1. This approval followed promising results from clinical trials such as KEYNOTE-355, which demonstrated that pembrolizumab plus chemotherapy significantly improved progression-free survival (PFS) compared to chemotherapy alone. The benefit was particularly pronounced in patients with PD-L1-positive tumors, emphasizing the role of PD-L1 as a predictive biomarker for immunotherapy response [10].

In addition to pembrolizumab, atezolizumab (an anti-PD-L1 monoclonal antibody) has also been tested in combination with chemotherapy, with the IMpassion130 trial showing that atezolizumab plus nab-paclitaxel improved overall survival (OS) in patients with metastatic TNBC who expressed PD-L1. These results have led to the approval of atezolizumab in combination with chemotherapy for PD-L1-positive TNBC [19].

PD-L1 (programmed death-ligand 1) plays a crucial role in the immune evasion mechanisms of tumors, including triple-negative breast cancer (TNBC). PD-L1 is an immune checkpoint protein that binds to the PD-1 receptor on T cells, leading to the suppression of T cell activation and proliferation. In normal immune function, this interaction helps to maintain self-tolerance and prevent autoimmune reactions by downregulating immune responses against healthy tissues. However, many cancer cells, including those in TNBC, exploit this pathway to evade immune surveillance and promote tumor progression [20].

ICIs’ mechanism of action aims at disrupting the PD-1/PD-L1 interaction, thereby restoring T cell activity against tumor cells [21,22]. Understanding the expression patterns of PD-L1 in TNBC, as well as its correlation with other clinical and pathological parameters, is critical for optimizing treatment strategies and improving patient outcomes.

This article aims to investigate the role of PD-L1 expression in triple-negative breast cancer, with particular emphasis on its correlation with various clinicopathological features, treatment responses, and HER2 expression. Additionally, we seek to evaluate potential differences in PD-L1 expression between primary tumors and residual tumors following neoadjuvant therapy, which can lead to insights for guiding everyday decision-making when choosing FFPE blocks for PD-L1 evaluation for TNBC.

## 2. Materials and Methods

Retrospectively, 88 newly diagnosed cases of triple-negative breast cancers, operated in Complex Oncological Center—Plovdiv, Bulgaria for the time period 2021–2023, were reviewed. Data about age, size, stage, lymph nodes status, and neoadjuvant treatment regimen were collected. Stage was evaluated according to the eighth edition of the American Joint Committee on Cancer (AJCC) [23].

All patients’ histological slides were evaluated by two pathologists in order to collect the following pathological parameters: histological subtype, grade, mitotic count per 10 HPFs, presence of perineural and lymphovascular invasion, and concentration of tumor-infiltrating lymphocytes (TILs). Grading was performed using WHO recommendations, numerically scoring from 1 to 3 the following morphological characteristics: tubular formation (>75%—1 point, 10–75%—2 points, less than 10%—3 points), nuclear pleomorphism (<1.5 times bigger than the size of nucleus of benign epithelial cell—1 point, 1.5 to 2 times larger—2 points, more than 2 times larger—3 points) and mitotic count per 10 HPFs in the hotspot area (0–4 mitoses—1 point, 5–9 mitoses—2 points, 10 or more mitoses—3 points). Tumors with scores in the range of 3–5 points were classified as grade 1, 6–7 points—as grade 2, and 8–9 points—as grade 3 [24]. Mitotic count was evaluated on microscopes with field number 22; thus, 10 HPFs corresponded to 2 mm^2^. To determine the TIL count, we used the scoring recommendations of the international consensus [25]. One representative slide per tumor was used and the area occupied by mononuclear inflammatory cells over total intratumoral stromal area was given in percentage. The results were rounded to the nearest 5%. In instances where a heterogeneous distribution of tumor-infiltrating lymphocytes (TILs) was present, the mean concentration across the entire slide was reported, rather than concentrating on hotspot areas. The evaluation excluded intratumoral TILs, TILs surrounding ductal carcinoma in situ (DCIS), and those located outside the tumor borders.

Of all the patients, 48 underwent 4 to 8 courses of neoadjuvant chemotherapy (NACT), 22 of which underwent a combination of neoadjuvant immunotherapy with pembrolizumab (NACT + IT). In these cases, the histological slides from the following surgical material were reviewed in order to establish the pathological response. We used the ICCR Invasive Carcinoma of the Breast in the Setting of Neoadjuvant Therapy Histopathology Reporting Guide [26] and the MD Anderson Residual Cancer Burden Calculator to estimate the residual cancer burden (RCB) and the residual cancer burden class. When residual disease was present, the following pathological parameters were also collected: largest tumor diameter, grade, mitotic count, perineural and lymphovascular invasion, and TILs count.

All immunohistochemical stains were performed on Ventana BenchMark Ultra (Roche Tissue Diagnostics (Oro Valley, AZ, USA)). One representative formalin-fixed paraffin embedded (FFPE) tissue block from every primary tumor was immunohistochemically tested for HER2 and Ki67 proliferation index, using the Ventana Ultra View visualization system and pre-diluted VENTANA anti-HER2/neu (4B5) Rabbit Monoclonal Primary Antibody and CONFIRM anti-Ki-67 (30-9) Rabbit Monoclonal Primary Antibody, respectively. HER2 expression was evaluated based on the ASCO/CAP 2023 evaluation algorithm [27]. Tumors with faint incomplete membrane staining in ≤10% of tumor cells or no staining were IHC scored as 0, tumors with weak to moderate incomplete membrane staining in >10% of tumor cells were IHC scored as 1+, and tumors with weak to moderate complete membrane staining observed in >10% of tumor cells were IHC scored as 2+. When there were cases with 2+ immunohistochemical staining, dual in situ hybridization was performed.

PD-L1 expression was tested on representative FFPE blocks from both primary and residual tumor, if there was such expression. The Dako 22C3 Clone and Ventana OptiView Visualization systems were used according to a recommended protocol. PD-L1 expression score was evaluated on immune and tumor cells, using the combined positive score (CPS) [28]. CPS was estimated as the number of PD-L1 staining tumors and mononuclear inflammatory cells divided by the total number of viable tumor cells. Linear membrane staining of any intensity in the tumor cells and cytoplasmic or membrane staining of any intensity in the inflammatory cells was taken into consideration. DCIS and areas with necrosis were not included in the evaluation. Cases with CPSs more or equal to 10 were considered positive and will hereafter be referred to in this article as “PD-L1-positive”; cases with CPSs less than 10 were considered negative and will be referred to as “PD-L1-negative”.

For statistical analysis IBM SPSS Statistics 19 was used. Chi-squared or Fisher’s exact tests were used to compare percentages between categorical variables. All of the variables, with the exception of age, were unequally distributed. The relationship between variables was analyzed by Spearman’s rank correlation coefficient (rho). The Mann–Whitney U test was used to compare means. Results were considered statistically significant if the *p*-value was below 0.05.

## 3. Results

### 3.1. Clinicopathological Findings

The age of the patients ranged from 32 to 88 years, with a mean age of 61.42 years. In total, 60 patients were diagnosed via core needle biopsy, 26 through excisional biopsy, and 2 cases of metastatic axillary lymph nodes were surgically removed. The largest tumor diameter varied between 5 mm and 100 mm, with a mean diameter of 31.55 mm. For cases involving excisional biopsy, tumor size was obtained from the pathological report, while in the remaining instances, data were derived from imaging studies.

Regarding the T stage, 78.2% were diagnosed at earlier T stages (T1 and T2), with T2 being the most prevalent at 50.6%, whereas T3 and T4 stage were less frequently observed, accounting for 8% and 13.8% of the cases. Almost half of the patients (47.7%) presented with positive nodal status on imaging studies at the time of the diagnosis.

Histologically, 83 cases were classified as carcinoma of no special type (NST), while 1 case was identified as a pleomorphic lobular carcinoma. Three cases exhibited apocrine features, and one case was categorized as a metaplastic carcinoma of the spindle cell variant. In terms of grading, no well-differentiated carcinomas were observed; 22.7% were moderately differentiated (G2) and 77.2% were poorly differentiated. The mean mitotic count was 10.32 mitoses per 10 high-power fields (HPFs), with a range of 2 to 41 mitotic figures. Lymphovascular invasion was observed in 13 cases (14.8%), while perineural invasion was identified in 7 cases (8%).

We evaluated TILs in a continuous manner and we further subdivided the cases into three predefined groups—with a low concentration of TILs (1–19%), with moderate TIL concentration (20–49%), and with high TIL concentration (50% or more). The mean count of tumor-infiltrating lymphocytes (TILs) among the patients in this study was 33.86%, with a range from 5% to 80%. A low TIL concentration was found in 19 (21.6%) of the samples, moderate—in 41 (46.6%), and high—in 28 (31.8%).

### 3.2. Immunohistochemical Findings

HER2-low expression was identified in 38.6% of the cases, with 6.8% classified immunohistochemically as 2+ but subsequently no amplification was found on dual in situ hybridization (DISH). The remaining cases within this category were scored as 1+ on immunohistochemistry. Additionally, 61.4% of the samples were categorized as HER2-negative, of which 42% exhibited no immunohistochemical signal, while 19.3% demonstrated a faint signal insufficient for a 1+ classification, thereby categorizing them as ultra-low expressors, which are generally regarded as indicating a negative reaction.

The mean Ki67 proliferation index was 58%, with a range from 10% to 90%. The majority of cases (95.5%) exhibited a proliferative index of 15% or more, with 78.4% of these cases showing a Ki67 index greater than 50%.

### 3.3. PD-L1 Expression

**Figure 1:**
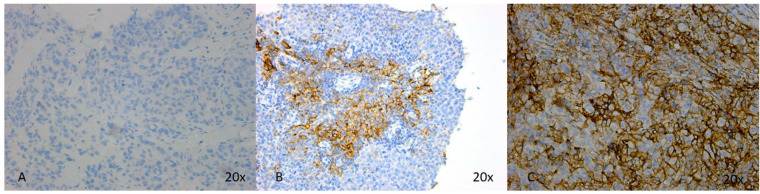
PD-L1 expression among TNBC. (**A**) Negative expression for PD-L1, estimated as CPS = 0; (**B**) positive expression for PD-L1, estimated as CPS = 20; (**C**) positive expression for PD-L1, estimated as CPS = 80.

All 88 primary tumors were tested for PD-L1. A total of 44.3% of them were classified as positive with a CPS equal or greater than 10. Of them, 9.1% showed a CPS equal or greater than 50. Null PD-L1 expressions were observed in 29.5% of the negative cases, the remaining scored with a CPS less than 10 (Figure 1).

Fourteen cases had previously undergone testing for PD-L1 expression at the time of diagnosis, which occurred 1 to 3 years prior to the current study. In thirteen of them, a CPS 10 or more was found. Among these, two cases were initially positive for PD-L1. However, during the ongoing study, their expression levels were estimated as less than 10. We subsequently retested PD-L1 expression using deeper sections from the formalin-fixed, paraffin-embedded (FFPE) blocks, but the results continued to be negative. The time from the previous test assessment and the present one for these two cases was one year and one year and four months, respectively.

In thirty-two cases, residual tumor was present following neoadjuvant treatment or surgery and these cases were subsequently tested for PD-L1 expression. Seven of these patients were evaluated for surgical resection after the biopsy, eighteen underwent NACT, and seven—NACT + IT. Among them, 28.9% were classified as positive, with a Combined Positive Score (CPS) of 10 or greater. Notably, 6.3% of these cases exhibited a CPS of 50 or higher. Null PD-L1 expression was observed in 28.1% of the negative cases, while the remaining cases were scored with a CPS of less than 10.

The mean PD-L1 CPS among the primary TNBC was 13.42, while among the residual tumors, it was 12.85. Although there was large difference in the PD-L1 mean score on residual tumors between patients who underwent NACT alone and patients, who underwent NACT + IT, 7.89 vs. 23.86, it was of no statistical significance (Mann–Whitney U = 56, *p* = 0.666; Effect size r = 0.862).

We identified a significant difference in positive PD-L1 expression between primary tumor biopsies and resection specimens, with 52.9% of initially positive cases losing their PD-L1 expression. Conversely, among previously negative cases, only one (6.7%) exhibited positive transformation (Pearson Chi-square = 6.432, df = 1, *p* = 0.011; Effect size phi = 0.448, Observed power = 0.769; Fisher’s exact test *p* = 0.018) (Table 1).

In a subgroup analysis, of the seven patients who underwent surgical treatment without neoadjuvant systemic therapy, no discordance in PD-L1 expression was observed. Specifically, two cases demonstrated positive PD-L1 expression in both the core needle biopsy and the surgical specimen, while five cases exhibited negative PD-L1 expression in both samples.

Among the seven patients who received neoadjuvant chemotherapy combined with immunotherapy (NACT + IT), all had a CPS above 10 in the primary tumor biopsy. However, 57.2% of these patients showed negative PD-L1 expression in the residual tumor samples. In addition, among the eighteen patients who underwent neoadjuvant chemotherapy alone (NACT), 62.5% experienced a change from positive to negative PD-L1 expression post-treatment, while one case (10%) exhibited the opposite change.

### 3.4. Correlation Between PD-L1 Expression and Clinicopathological Features

PD-L1 positive expression was more frequently observed in patients below the age of 50. However, this difference did not reach statistical significance (Mann–Whitney test U = 519, *p* = 0.104, Effect size r = 0.2). Among PD-L1 negative cases, a lower T stage was noted in a slightly larger proportion, whereas among PD-L1 positive cases, there was a higher proportion of T4 presentations. Nonetheless, these differences were not statistically significant (Chi-square = 5.575, df = 3, *p* = 0.134, Effect size phi = 0.253, Observed power = 1).

Although it did not reach statistical significance, PD-L1-positive triple-negative breast cancers were more commonly associated with lymph node metastases (Chi-square = 5.355, df = 1, *p* = 0.021; Fisher’s exact test *p* = 0.031, Effect size phi = 0.3, Observed power = 0.081). No correlation was found between tumor grade and positive PD-L1 expression. While a slightly higher mitotic activity was observed among PD-L1-positive cases, this finding was not statistically significant.

A statistically significant correlation was established between PD-L1 expression and the concentration of tumor-infiltrating lymphocytes (TILs). The mean concentration of TILs among positive cases was 41.79%, compared to 27.55% among negative tumors (Mann–Whitney U = 559.5, *p* = 0.001, Effect size r = 0.4). This correlation was further confirmed by subsequent correlation analysis (Spearman Correlation Coefficient r = 0.396, *p* < 0.001) (Table 2).

### 3.5. Correlation Between PD-L1 and Immunohistochemical Parameters

**Figure 2:**
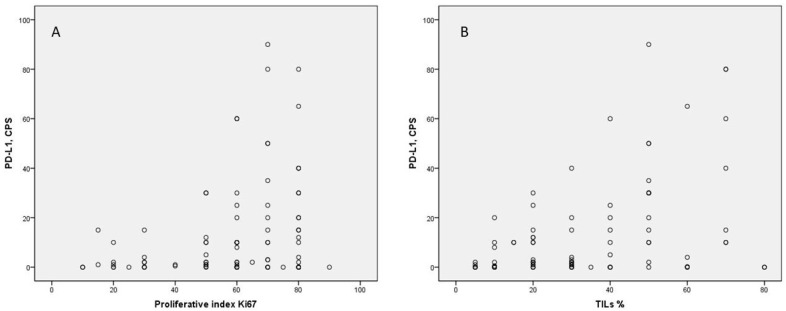
Correlation between PD-L1 and TILs, rho = 0.396, *p* < 0.001 (**B**) and PD-L1 and Ki-67, rho = 0.236, *p* = 0.027 (**A**).

No correlation was found between PD-L1 and HER2 expression. In relation to the Ki-67 proliferative index, higher expression levels were observed in PD-L1-positive tumors, with the majority of cases exhibiting Ki-67 levels exceeding 50%. A statistically significant difference was noted between the median proliferative indexes of the two groups, with a median of 64.49 in the PD-L1 positive cases compared to 52.86 in the negative cases (Mann–Whitney U = 670.5, *p* = 0.015, Effect size r = 0.3). These findings were further supported by correlation analysis, which confirmed a positive correlation between the two parameters (Spearman correlation coefficient = 0.236, *p* = 0.027) (Table 2), (Figure 2).

### 3.6. Pathological Response to Neoadjuvant Treatment

Overall, improved treatment outcomes were observed in patients receiving combined immunotherapy and chemotherapy compared to those undergoing chemotherapy alone, with a mean residual cancer burden (RCB) score of 0.94753 in the former group versus 2.55148 in the latter (Mann–Whitney U = 89, *p* = 0.003, Effect Size r = 0.5).

No significant differences in mean RCB scores were found between PD-L1-positive and -negative cases.

**Figure 3:**
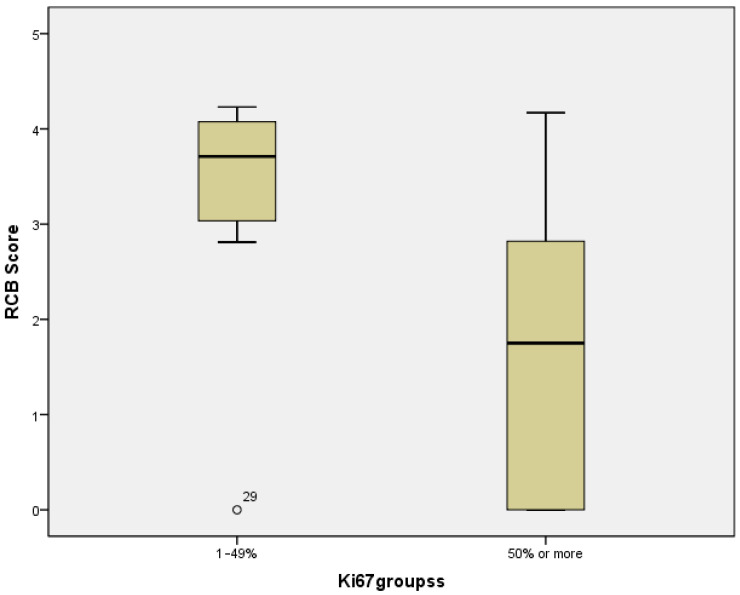
Ki-67 by Residual Cancer Burden Score strata. The middle line indicates the median value, and the bottom and top edges of the box represent the 25th and 75th percentiles, respectively. The whiskers extend to the most extreme data points that are not considered as outliers, and the outliers are plotted individually using circle sign. The mean RCB score for highly proliferative tumors was 1.59509, compared to 3.16486 in cases with a Ki-67 index below 50% (Mann–Whitney U = 53, *p* = 0.022, Effect size r = 0.4).

A negative correlation was found between RCB score post-neoadjuvant treatment and Ki67 (Spearmen’s correlation coefficient rho = −0.448, *p* = 0.004), which means the higher the proliferation index is, the better the outcome of the treatment is. A statistically significant better treatment outcome was noted in cases with a proliferative Ki-67 index exceeding 50%. The mean RCB score for highly proliferative tumors was 1.59509, compared to 3.16486 in cases with a Ki-67 index below 50% (Mann–Whitney U = 53, *p* = 0.022, Effect Size r = 0.4) (Figure 3). When the threshold was decreased, a statistically significant difference remained evident between cases with Ki-67 levels below and above 30%, with mean Residual Cancer Burden (RCB) scores of 3.22483 and 1.63068, respectively (Mann–Whitney U = 45, *p* = 0.027, Effect size r = 0.4). In contrast, when the threshold was further lowered to 20% or less, no statistically significant difference was observed.

Furthermore, no predictive significance was identified for tumor-infiltrating lymphocyte (TIL) concentration concerning treatment outcome.

Moreover, a favorable pathological response, defined as a residual cancer burden (RCB) classification of 0 or 1, was observed more frequently among HER2-negative cases, with a prevalence of 54.5%, compared to 25% in HER2-low tumors (Chi-square = 4.425, df = 1, *p* = 0.035, Effect size phi = 0.3, Observed power = 0.627; Fisher’s exact test, *p* = 0.048). Additionally, a statistically significant difference in mean RCB scores was identified between the two groups, with HER2-low cases exhibiting a mean score of 2.76657, in contrast to 1.38692 for HER2-negative cases (Mann–Whitney U = 93, *p* = 0.010, Effect size r = 0.4) (Figure 3).

We also performed a stepwise multiple linear regression analysis to evaluate the impact of Ki-67 proliferative index, HER2 expression, concentration of TILs%, and PD-L1 expression on the outcome of neoadjuvant treatment, represented by the Residual Cancer Burden Score. Ki-67 expression turned out to be good predictor of treatment outcome (r^2^ = 0.18, F = 8.198, *p* = 0.007). The other variables were excluded from the regression model. We further investigated the correlation between HER2 expression and the other factors and it turned out that there is a strong negative correlation between HER2 immunohistochemical status and Ki67 proliferative index (Spearman’s correlation coefficient rho = −0.321, *p* = 0.002).

## 4. Discussion

Triple-negative breast cancer (TNBC) represents a biologically diverse subgroup of mammary gland carcinomas, characterized by poorer prognosis and fewer treatment options compared to other breast cancer subtypes. However, recent advancements in immunotherapy have demonstrated efficacy in treating TNBC, both in early-stage and metastatic settings [29]. Currently, after the results of the KEYNOTE-355 trial, the only approved immune checkpoint inhibitor (ICI) for metastatic TNBC with PD-L1 CPS expression higher or equal to 10 is pembrolizumab in combination with chemotherapy [10]. In the neoadjuvant setting, there are two approved treatment possibilities: pembrolizumab plus chemotherapy and atezolizumab plus chemotherapy as neoadjuvant treatment, regardless of PD-L1 expression [30,31].

Atezolizumab is an inhibitor of PD-L1, while pembrolizumab targets PD-1. PD-1 is a cell surface receptor found on the membrane of T lymphocytes, whereas PD-L1 is a ligand that acts as a co-receptor, binding to PD-1. Under normal physiological conditions, the interaction between PD-1 and PD-L1 serves to regulate immune responses, preventing autoimmunity and excessive immune activation [32]. However, certain tumor cells exploit this pathway as an escape mechanism by overexpressing PD-L1 on their surface. This expression effectively inhibits the host’s antitumoral immune response, allowing the tumor to evade immune detection and destruction [33].

To date, PD-L1 expression is regarded as the most reliable biomarker for predicting the effect of immunotherapy. In the context of triple-negative breast cancer (TNBC), several assays are utilized, with the most implemented being SP142, Ventana, and 22C3 from Dako. Each of them has a specific scoring system—immune count for SP142, focusing exclusively on the tumor area occupied by PD-L1-expressing tumor-infiltrating immune cells (IC), and Combined Positive Score for 22C3, considering positive expression in both tumor and immune cells (CPS) [34]. Each drug available on the market is accompanied by a specific PD-L1 diagnostic assay, which is crucial for ensuring the effective and safe administration of the therapy [29]. However, these complexities may predispose to diagnostic challenges.

Several studies have compared the expression rates for PD-L1 using different antibodies. Sigurjonsdottir et al. (2023) found that the SP142 assay was more effective at identifying tumors positive for the 22C3 clone than the 22C3 assay was at detecting SP142-positive tumors [35]. Similarly, Ivanova et al. (2023) demonstrated that DAKO 22C3 and Ventana SP263 assays can be reliably used interchangeably when assessing the Combined Positive Score (CPS). However, when evaluating the Immune Cell (IC) score, the 22C3 and SP263 assays should not be substituted for the SP142 assay [36].

Another diagnostic challenge in PD-L1 testing is the selection of the most appropriate formalin-fixed, paraffin-embedded (FFPE) tissue block. Several factors can influence PD-L1 expression, including issues during the preanalytical phase, such as prolonged cold ischemia time, tissue processing, and decalcification. Studies have shown that antibodies binding to the external domain of PD-L1 (e.g., 22C3, 28-8) are more sensitive to prolonged cold ischemia than those targeting the internal domain of the receptor (e.g., SP142, SP263) [37]. This suggests that core needle biopsy specimens, which typically undergo better fixation, may be more suitable for PD-L1 testing. However, given the heterogeneous expression of PD-L1 across tumor tissues, it may also be more reliable to use resected tissue to reduce the risk of misinterpretation.

Various studies examining PD-L1 expression in core needle biopsies versus corresponding surgical specimens have reported higher positive expression rates when testing is conducted on resection material [29,38,39]. There is substantial evidence indicating that PD-L1 expression is generally lower in metastatic lesions compared to primary tumors. This discrepancy can be attributed to alterations in the tumor microenvironment that occur during metastasis [40,41].

There is small number of studies in the English literature that compare PD-L1 expression in primary versus residual tumors following neoadjuvant therapy. The first one, conducted by Vasiliki Pelekanou et al., reported a significant decrease in PD-L1 expression after neoadjuvant therapy [42]. Ji Won Woo et al. found negative to positive conversion in 22% of triple-negative breast carcinomas, following neoadjuvant platinum-based treatment [43]. Notably, one more study by Grandal et al. found that less than 20% of pretreated residual triple-negative breast cancer (TNBC) cases exhibited PD-L1 positivity, which is less than the reported in the literature rates for PD-L1 positivity among TNBC [44,45].

There are also studies indicating that PD-L1 expression is positively influenced by neoadjuvant chemotherapy in various other cancers, like esophageal squamous cell carcinoma and cervical squamous cell carcinoma [46,47]. However, regarding lung non-small cell lung carcinoma, the clinical data suggest that PD-L1 expression is mostly unchanged after neoadjuvant chemotherapy [48,49].

In our study, among patients who did not receive neoadjuvant treatment, we observed complete concordance in PD-L1 expression between core needle biopsies and corresponding resection specimens. In contrast, among patients who received combined neoadjuvant immunotherapy and chemotherapy, 57.2% exhibited a loss of PD-L1 expression, while 62.5% of patients treated with neoadjuvant chemotherapy alone demonstrated a transformation from positive to negative PD-L1 expression. These findings may provide a compelling reason to introduce immune checkpoint inhibitors (ICIs) as a frontline therapy, thereby optimizing the potential benefits of this treatment. However, the existing data are very limited and extensive investigation in this direction is needed for better understanding the impact of neoadjuvant therapy on PD-L1 expression in the field of TNBC.

One potential mechanism underlying the downregulation of PD-L1 expression is the ubiquitination of the PD-L1 receptor by E3 ubiquitin ligases, such as STUB1 [50], Cullin3-SPOP [51], and β-TrCP (β-transducin repeat-containing protein) [52]. Additionally, another mechanism for the ubiquitination and subsequent degradation of the PD-L1 receptor may involve the activation of the cyclin D1-CDK4 pathway [51]. A possible explanation for the failure to achieve a complete pathological response and the observed decrease in PD-L1 expression following treatment could be the activation of the cyclin D1-dependent pathway. Another potential explanation for the reduced expression of PD-L1 following treatment is the activation of autophagic degradation through the HIP1R and PKCα/GSK3β/MITF pathways [53]. This may serve as an escape mechanism, particularly in cases where immune checkpoint inhibitors (ICIs) are administered in the neoadjuvant setting. Both overexpression and underexpression of PD-L1 can undermine the efficacy of immunotherapy [54].

Another pitfall when selecting FFPE blocks is the age of the tumor specimen. Evidence suggests that the antigenicity of PD-L1, particularly for the 22C3 and 28-8 clones, can significantly decrease within three years due to the degradation of the glycan component of the epitope in the extracellular domain of PD-L1 [55].

Among the cases we evaluated, fourteen had also been assessed at the time of diagnosis; two of these lost their PD-L1 positivity within one year and one year and four months, respectively. This indicates that antigenicity may diminish in some cases within a period shorter than three years. These findings, along with the observation that PD-L1 expression was lost in approximately half of the cases that received neoadjuvant chemotherapy and immunotherapy, highlight several important practical considerations for clinical practice. The 22C3 PD-L1 clone is currently the only approved companion diagnostic for the use of pembrolizumab in advanced triple-negative breast cancer (TNBC). However, standard treatment protocols do not require a rebiopsy of metastatic lesions following progression, which often necessitates the use of archival materials for PD-L1 testing. Given this, it may be clinically significant to routinely perform reflex testing for PD-L1 expression on primary core needle biopsies. Without this approach, PD-L1 expression could be underestimated in a substantial number of cases.

In addition to its predictive value, researchers are investigating the prognostic significance of PD-L1 expression in breast carcinomas. Current data on this topic are controversial, with some studies reporting a negative prognostic value of PD-L1, others indicating a positive correlation, and still others finding no significant association [56,57,58,59,60,61]. A recent study by Nataša Medić-Milijić et al. suggested that the concurrent testing of multiple biomarkers may hold greater prognostic significance than PD-L1 alone. The authors identified a “high-risk” profile for triple-negative breast cancer (TNBC), characterized by low expression levels of PD-L1 and androgen receptor (AR), alongside high expression levels of epidermal growth factor receptor (EGFR) and Ki67 [62]. In our study, we observed that PD-L1 positive expression was associated with a slightly younger patient demographic, positive lymph node status, and a high proliferative index. Given that elevated Ki-67 expression is linked to a poorer prognosis, it can be assumed that PD-L1 expression may similarly correlate with adverse outcomes. Conversely, a high Ki-67 index is associated with increased rates of pathological complete response (pCR), a favorable prognostic indicator for overall survival.

We also found that positive PD-L1 expression is connected with higher concentrations of tumor-infiltrating lymphocytes, which is consistent with previous data in the literature [63,64]. To date, there are no existing criteria regarding what TILs count should be considered low or high. For instance, the German Cancer Group considers < 10% low, 10–59% intermediate, and 60% or more high [65]. De Jong et al. in their study distinguish patients with high sTILs (≥75%) from those with low sTILs (<30%) [66], while other authors like Hendry et al. and D.Y. Lee et al. prefer to divide breast carcinomas into lymphocytes-predominant BC (with 50% or more stromal tumor-infiltrating lymphocytes) and non-LPBC [67]. In another review article, focusing on the predictive value of TILs for achieving pCR after neoadjuvant therapy, the predominant cutoff value for high TILs concentration was 50% among the studies included [68]. In our study, we considered the concentration of stromal TILs over 50% as high.

In the past year, another emerging treatment option for triple-negative breast carcinoma has become available: trastuzumab–deruxtecan, an antibody–drug conjugate that has demonstrated efficacy in tumors with low HER2 expression [69]. HER2-low expressing tumors are characterized by an immunohistochemical signal classified as 1+ or 2+, with no detected amplification on in situ hybridization [70]. Our study aimed to investigate any potential correlation between PD-L1 expression and HER2 expression within the context of triple-negative breast cancer to inform future treatment decision-making. However, we found no correlation, which was anticipated, given the distinct biological nature of these two markers.

There is ongoing research aimed at identifying clinical and pathological markers that predict treatment outcomes in patients with triple-negative breast cancer (TNBC). Among these, tumor-infiltrating lymphocyte (TIL) concentration has been identified as a promising predictor [71,72]. TILs typically represent a diverse population of immune cells, including cytotoxic CD8+ T lymphocytes, CD4+ T-helper cells, FOXP3+ regulatory T cells, macrophages, natural killer cells, and dendritic cells [73]. High concentrations of CD8+ cytotoxic T cells (CTLs), as well as a high CTLs to FOXP3+ regulatory T cells ratio, have been identified as key predictive factors for favorable treatment outcomes [74,75]. In our study, we did not observe any predictive value related to TIL concentrations.

There are multiple studies investigating the potential role of Ki-67 in predicting treatment outcome in TNBC. The existing data are controversial, with some authors finding a correlation between high Ki-67 levels and pCR rates [76,77,78], and others not finding any correlation [79,80,81]. The issue of determining an appropriate cutoff value for Ki-67 is another challenge in its use as a biomarker. In a review by Nadine S. van den Ende et al., which addressed this issue, it was noted that in most studies identifying a predictive value for Ki-67, a cutoff of 50% or higher was commonly used [68]. In our study, we found a significant predictive value of Ki-67 levels exceeding 50% for treatment outcomes. The same was confirmed for Ki-67 expression over 30%.

Another potential predictive biomarker for the efficacy of immune checkpoint inhibitors (ICIs) is tumor mutation burden (TMB). High TMB has been associated with improved responses to ICIs across a variety of solid tumors [82]. Additionally, tumors with deficient DNA mismatch repair (dMMR) systems also demonstrate a better response to ICI therapy [83]. Recent studies, including data from the KEYNOTE-016, KEYNOTE-164, KEYNOTE-012, KEYNOTE-028, and KEYNOTE-158 trials, have shown that immunotherapy is particularly effective in tumors exhibiting microsatellite instability-high (MSI-H) or dMMR status, including those from the colon, cervix, endometrium, esophagus, stomach, biliary tract, and breast [84].

Recent studies have demonstrated that the Ataxia telangiectasia mutated (ATM) protein, a key component of the mechanisms involved in homologous recombination and DNA repair, can lead to the downregulation of PD-L1 expression through the suppression of tumor necrosis factor-alpha (TNF-α) [85]. This suggests that deficiencies in DNA repair mechanisms may serve as a potential predictive marker for response to immune checkpoint inhibitors (ICIs). Furthermore, these findings imply that treatment with PARP or ATM inhibitors could enhance the efficacy of ICIs [86].

The predictive significance of HER2 expression in the context of TNBC remains underexplored. Some studies suggest that there is no significant difference in pathological response to neoadjuvant treatment between HER2-low and HER2-negative cases [87,88]. However, our study revealed a favorable pathological response, defined as a residual cancer burden classification of 0–1, in 54.5% of HER2-negative cases, compared to 25% among HER2-low expressors. Univariate analysis showed statistically significant difference in mean residual cancer burden scores between the HER2-negative and HER2-low groups, but this finding was not further supported by multiple regression analysis. The reason for that was the estimated strong association between HER2-low expression and the Ki-67 proliferative index. Further investigation in this area is warranted, as it could significantly influence treatment decision-making for patients with TNBC.

Our study has several limitations. First, the patient cohort was relatively small. Second, as with many other studies, there were no standardized criteria for scoring methods or cutoff values for certain markers, such as Ki-67 and TILs. Third, the effect of treatment was assessed in a generalized manner, and due to the small cohort size, we were unable to perform subgroup analyses based on the different therapeutic regimens employed.

Although this study is relatively simple in design, the researchers believe that even simple studies can make significant contributions to improving patient treatment decisions. Our study presents some pragmatic insights. To date, PD-L1 expression in triple-negative breast cancer (TNBC) is routinely assessed only in the adjuvant setting; however, rebiopsy of patients with disease progression is not mandatory. Considering our finding that approximately half of the PD-L1-positive cases lost expression following neoadjuvant chemotherapy and the fact that FFPE antigenicity can degrade within a period of less than three years, we propose that reflex testing for PD-L1 on primary TNBC biopsies should be considered as a potential standard of care in the future.

## 5. Conclusions

In conclusion, this study provides valuable insights into the clinicopathological characteristics and PD-L1 expression in newly diagnosed triple-negative breast cancer (TNBC) patients. These findings highlight a significant discordance in PD-L1 expression between primary tumors and residual tumors following neoadjuvant treatment, with a notable proportion of patients experiencing a loss of PD-L1 positive expression. The data suggest that factors such as age, lymph node status, and tumor-infiltrating lymphocyte concentration may influence PD-L1 expressions. Furthermore, the study underscores the necessity for routine reflex testing of primary core needle biopsies for PD-L1, especially in the context of neoadjuvant chemotherapy and immunotherapy, to avoid underestimating PD-L1 expression in clinical practice.

Additionally, the results of this study suggest that the Ki-67 proliferation index might have predictive significance for treatment outcome in TNBC.

Overall, this research adds to the existing knowledge regarding the complexities of PD-L1 as a biomarker in breast cancer, emphasizing the need for further investigation into its prognostic implications and therapeutic relevance.

## Figures and Tables

**Table 1:** PD-L1 expression on primary tumors vs. PD-L1 expression on residual tumors.

		PD-L1 Expressions on Residual Tumors	
		CPS < 10	CPS ≥ 10
**PD-L1 Expression on Primary Tumors**	**CPS < 10**	14 (93.3%)	1 (6.7%)
	**CPS ≥ 10**	9 (52.9%)	8 (47.1%)

**Table 2:** Clinicopathological characteristics of PD-L1-positive and PD-L1-negative cases. Statistically significant differences are bolded.

Overall (*N* = 88)	PD-L1+ (*N* = 39)	PD-L1− (*N* = 49)	*p*-Value
Age	57.81 (32–88)	63.84 (37–88)	0.104
Stage			0.134
T1	7 (18.4%)	17 (34.7%)	
T2	21 (55.3%)	23 (46.9%)	
T3	2 (5.3%)	5 (10.2%)	
T4	8 (21.1%)	4 (8.2%)	
Positive lymph nodes	**24 (61.5%)**	**18 (36.7%)**	**0.081**
Grade			0.134
G2	6 (15.4%)	14 (28.6%)	
G3	33 (84.6%)	35 (71.4%)	
Mitotic count	11.33 (2–41)	9.51 (2–27)	0.161
TILs%	**41.79 (10–70)**	**27.55 (5–80)**	**0.001**
HER2-low	15 (38.5%)	19 (38.8%)	1.0
Ki-67%	**64.49 (15–80)**	**52.86 (10–90)**	**0.015**
Ki-67 ≥ 50	**36 (92.3%)**	**33 (67.3%)**	**0.008**

## Data Availability

Data are contained within the article.
